# Assessment of Glioblastoma Response in the Era of Bevacizumab: Longstanding and Emergent Challenges in the Imaging Evaluation of Pseudoresponse

**DOI:** 10.3389/fneur.2019.00460

**Published:** 2019-05-07

**Authors:** Octavio D. Arevalo, Carolina Soto, Pejman Rabiei, Arash Kamali, Leomar Y. Ballester, Yoshua Esquenazi, Jay-Jiguang Zhu, Roy Francisco Riascos

**Affiliations:** ^1^Department of Diagnostic and Interventional Radiology, McGovern Medical School, The University of Texas Health Science Center at Houston, Houston, TX, United States; ^2^Faculty of Medicine, Universidad Nacional de Colombia, Bogotá, Colombia; ^3^Department of Pathology and Laboratory Medicine, McGovern Medical School, The University of Texas Health Science Center at Houston, Houston, TX, United States; ^4^Vivian L. Smith Department of Neurosurgery, McGovern Medical School, The University of Texas Health Science Center at Houston, Houston, TX, United States

**Keywords:** pseudoresponse, bevacizumab, MRI imaging, perfusion-weighted imaging, MR spectroscopy, diffusion weighted imaging (DWI), susceptibility weighted imaging (SWI), diffusion tensor imaging (DTI)

## Abstract

Glioblastoma is the deadliest primary malignant brain neoplasm, and despite the availability of many treatment options, its prognosis remains somber. Enhancement detected by magnetic resonance imaging (MRI) was considered the best imaging marker of tumor activity in glioblastoma for decades. However, its role as a surrogate marker of tumor viability has changed with the appearance of new treatment regimens and imaging modalities. The antiangiogenic therapy created an inflection point in the imaging assessment of glioblastoma response in clinical trials and clinical practice. Although BEV led to the improvement of enhancement, it did not necessarily mean tumor response. The decrease in the enhancement intensity represents a change in the permeability properties of the blood brain barrier, and presumably, the switch of the tumor growth pattern to an infiltrative non-enhancing phenotype. New imaging techniques for the assessment of cellularity, blood flow hemodynamics, and biochemistry have emerged to overcome this hurdle; nevertheless, designing tools to assess tumor response more accurately, and in so doing, improve the assessment of response to standard of care (SOC) therapies and to novel therapies, remains challenging.

## Background

Glioblastoma (GBM) is the most common primary malignant brain neoplasm with an incidence of 4/100,000. GBM accounts for 54% of all glial tumors and 45% of all malignant central nervous system (CNS) tumors. Prognosis of patients with GBM is dismal with a median survival of 14–16 months, and a survival rate of < 30 and 10% at 2–5 years after the initial diagnosis, respectively (1). From the histological standpoint, GBMs are infiltrating glial tumors, displaying abnormal glial cells with variable morphology, high mitotic activity, microvascular proliferation and necrosis with pseudopalisading patterns. Microvascular proliferation and necrosis are two critical histologic features used for the differentiation between an anaplastic astrocytoma, WHO grade III and a glioblastoma, WHO grade IV (2). Early clinico-pathological studies demonstrated that the degree of microvascular proliferation, as a surrogate of tumor-driven neo-angiogenesis, correlated with survival in patients with high-grade glial tumors (3, 4). Subsequently, researchers proved that tumor enhancement on gadolinium-enhanced magnetic resonance imaging (MRI) of the brain was a reflection of the microvascular density and could be used as a biomarker of tumor activity and aggressiveness (5). For the past two decades, the presence and characteristics of enhancement patterns have been one of the cornerstones for the imaging diagnosis and determination of treatment responses of malignant brain tumors. However, its role has been debated, and some novel ancillary imaging biomarkers have been developed in the complex and ever-evolving neuroradiologist's armamentarium.

## Glioblastoma Treatment

The neuro-oncology team faces a unique challenge when treating patients diagnosed with GBM given the intrinsic biologic peculiarities of this tumor. Many factors, such as the permeability restrictions to the anti-tumor drugs posed by the blood brain barrier (BBB), the tumor cell population heterogeneity, the involvement of vital eloquent structures, and the adaptive nature of the tumor have contributed to the limited success of standard of care (SOC) and the failure of many experimental drugs. The current SOC treatment for newly diagnosed GBM has not changed since Stupp et al. published the results of the EORTC-NCIC trial in 2005 (6). The Stupp protocol consists of maximal safe surgical resection of the tumor, followed by concurrent fractionated radiotherapy and daily temozolomide for 6 weeks. After completion of the chemo-radiation, patients continue to receive 6 to 12 cycles of monthly adjuvant temozolomide with the addition of TTF.

Unfortunately, most of the GBM patients experience tumor recurrence between 6 and 9 months while on SOC treatments. Current guidelines for recurrent GBM recommend multi-disciplinary team care with therapies tailored to individual patient's unique situation including a combination of re-resection, re-radiation, Laser Interstitial Thermal Therapy (LITT), change chemotherapy to bevacizumab monotherapy or combination with other drugs plus TTF. Nevertheless, most of the treatment regimens have failed to prolong the overall survival rate in patients with progressive disease (7). Tumor treating fields therapy (TTF) is a novel therapeutic device that has emerged since 2011 for recurrent GBM and then in 2015 for newly diagnosed GBM (8, 9)

In recent years, researchers have identified molecular markers that help to classify GBMs into subgroups with different treatment response profiles and prognosis (10). For instance, gliomas harboring a mutation of the isocitrate dehydrogenase 1 or 2 (IDH 1/2) gene tend to have better survival and comparing those with the wild-type allele (11); or tumors with methylation of the O^6^-methylguanine-DNA-methyltransferase (MGMT) gene promoter are more sensitive to temozolomide/radiation therapies resulting in better survival rates (12, 13). The growing understanding of brain tumor genetics and signal transduction pathways abnormalities facilitates the creation of targeted therapies; some of them, with specific imaging response patterns.

## Role of MRI in Glioblastoma Diagnosis and Surveillance

MRI is the current standard for imaging evaluation of brain neoplasms for diagnosis and measurement of response, in both clinical practice and clinical trials. The required sequences of the current MRI protocol for the assessment of glioblastoma are three-dimensional T1-weighted images (3D-T1w), axial bi-dimensional T2-Fluid-attenuated inversion recovery (FLAIR) images, and axial bi-dimensional diffusion-weighted imaging (DWI) before intravenous gadolinium-based contrast agent (GBCA) administration. After contrast administration, the required sequences are axial bi-dimensional T2-weighted (T2w) images and three-dimensional T1-weighted images (3D-T1w) images (1).

As previously stated, the pattern of enhancement has been used as a surrogate of tumor grade and viability during the last two decades; nevertheless, when evaluating patients with GBM after treatment, the presence and degree of enhancement cannot be attributed exclusively to tumor neo-angiogenesis or high cell replication areas. The differential diagnosis list of increased parenchymal enhancement in patients with post-treatment GBM is broad and includes tumor-related and non-tumoral causes, such as the increase of the permeability of the BBB secondary to chemoradiotherapy, as well as superimposed ischemia or surgical trauma. In summary, all processes that alter the BBB permeability will modify the degree and extent of the enhancing area, regardless of the size and activity of the tumor (14). Given the heterogeneous nature of GBM cell populations, some areas of the tumor display an infiltrative growth pattern and may not enhance (15, 16). This tissue heterogeneity makes the current role of MRI on GBM evaluation even more challenging. With the advancement of imaging technologies, there is an increased research effort for the development of more accurate imaging techniques. Some of the advanced MRI sequences permit the evaluation of cellular variability, such as tumor perfusion hemodynamics, tissue chemistry and metabolism, and cellularity; nonetheless, they are not yet used in clinical practice.

## Evolution of Response Assessment Criteria in Glioblastoma

The poor prognosis of GBM patients has motivated researchers to develop more effective treatments during the last half-century; and therefore, the necessity of standardizing criteria for the semi-quantitative assessment of response to treatment was subsequently conceived. With the purpose of making trials outcomes comparable, Levin et al. developed the first criteria for the evaluation of patients undergoing chemoradiotherapy for malignant brain tumors in 1977 (17). These criteria included the assignment of a numeric score to variables such as findings in the neurological examination, electroencephalographic patterns, brain scintigraphy, and the presence of specific images on brain computerized tomography (CT). Based on the final score, the patients were classified into two main categories: tumor regression or tumor growth. Two years later, the World Health Organization (WHO) issued its first guideline on reporting results of cancer treatment. From the imaging standpoint, the WHO guidelines suggested measuring the perpendicular diameters of target lesions and quantifying the change of its product over serial imaging examinations. Although it was not focused on GBM (18), it was a milestone because of the introduction of the concept of complete response (CR), partial response (PR), progressive disease (PD), and no change.

In 1990, Macdonald et al. created a paradigm on the response assessment of supratentorial malignant gliomas by publishing criteria that considered CT/MRI findings as the keystone in light of the clinical findings and steroid use (19). Macdonald criteria established rigorous definitions for the main four response categories: CR (disappearance of all enhancing tumor on consecutive scans, at least 1 month apart, in the absence of steroids, and stable, or improved neurologic deficit), PR (≥50% reduction in size of enhancing tumor on consecutive scans, steroids dose stable or reduced, and clinically stable or improved), and PD (≥25% increase in size of enhancing tumor or any new tumor on imaging, or neurologically worse, and steroids stable, or increased). Additionally, it changed the “No change” category of the WHO classification for stable disease (SD) when none of the above criteria are met. Despite its robustness, the Macdonald criteria's “Achilles' heel” was its foundation on the enhancement as the unique imaging biomarker of tumor viability. The weaknesses of the Macdonald criteria became evident when changes in the BBB permeability secondary to non-tumoral processes led to misclassification of patients into the wrong response category based on the increase or decrease of the degree of enhancement.

## The Era of the Antiangiogenic Therapy

In 2009, the US Food and Drug Administration (FDA) approved the use of bevacizumab (BEV or Avastin®) for the treatment of recurrent GBM (20). BEV is a humanized monoclonal antibody directed to the isoform A of the vascular endothelial growth factor (VEGF). Its therapeutic effect is blocking the process of angiogenesis, one of the main features of GBM pathogenesis. Under the effect of BEV, the immature and friable vasculature of the tumor is stabilized, and the rate of microvascular proliferation and the BBB permeability decrease (21). These microstructural and functional changes are translated into a dramatic and almost immediate reduction in the tumor enhancement on MRI scans (22, 23). It was initially interpreted as overwhelming tumor response, but its clinical significance soon became controversial. In 2014, Gilbert et al. published the results of a phase III clinical trial on 637 newly diagnosed GBM patients comparing the SOC vs. SOC with BEV and detected a modest increase of the progression-free survival (PFS) rate without improvement in the overall survival (OS) rate (24). At the meantime, Chinot et al. reported their phase III clinical trial with 458 patients administering BEV plus SOC vs. SCO for newly diagnosed GBM patients and found that there was a slight improvement of the PFS with maintenance of quality of life without any impact on the OS (25). Both trials concluded that adding BEV for newly diagnosed GBM does not improve OS. One caveat is that in both trials, patients in SOC arms received BEV during GBM progression. It was evident that there was a dissociation between the traditional radiological response assessment and the clinical outcomes.

## The Ground-Breaking Concept of Pseudoresponse

**Figure 1:**
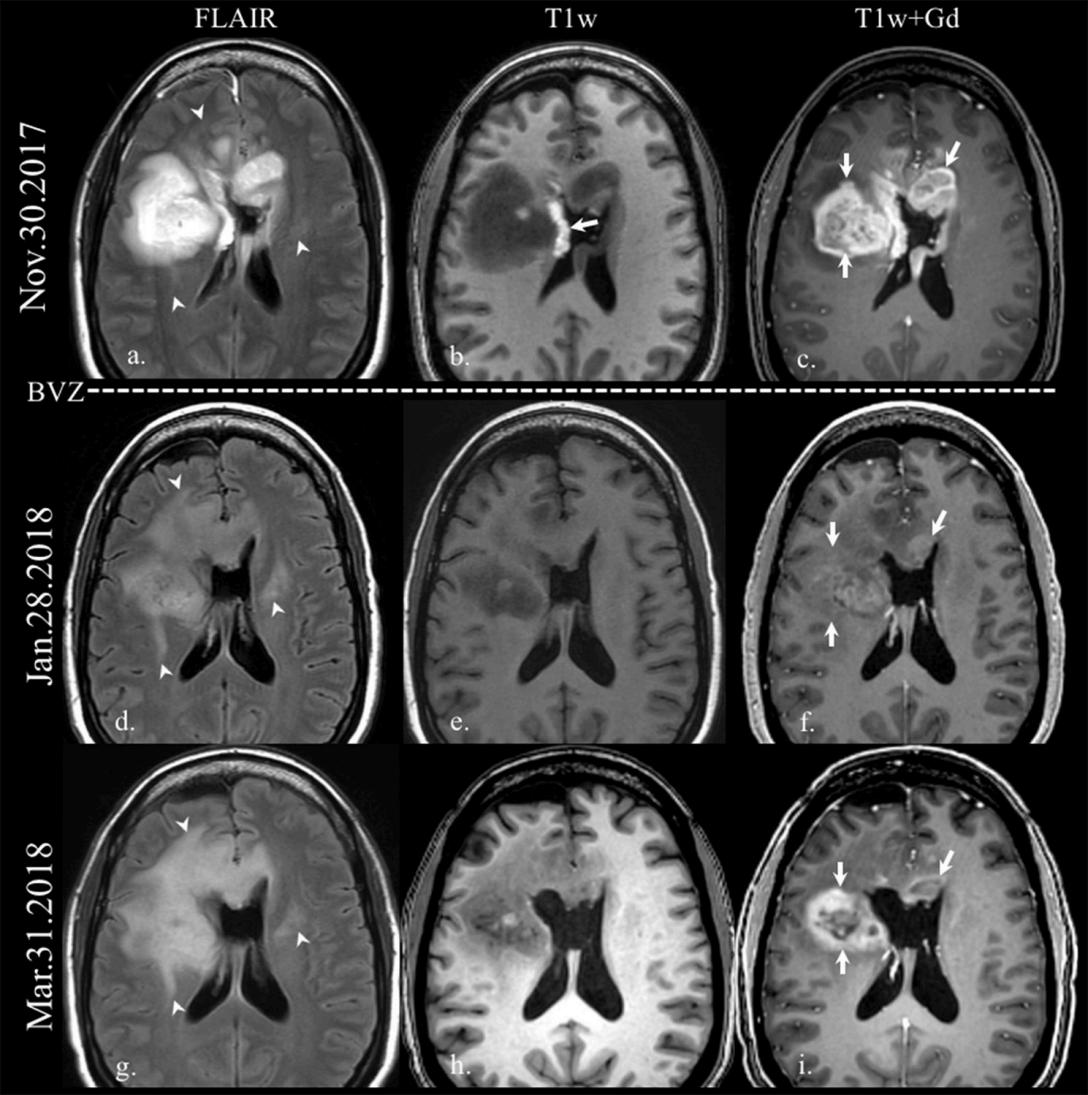
Enhancement in pseudoresponse. Brain MRI of a old patient with a GBM (IDH wild-type, MGMT status unknown) on the first recurrence after standard-of-care treatment. Axial FLAIR **(a,d,g)**, and axial T1w before **(b,e,h)** and after gadolinium administration **(c,f,i)** images are displayed. On the top row, the immediate postoperative scan after the second resection demonstrates residual enhancing tumor on the right frontal lobe and on the left aspect of the genu of the corpus callosum (arrows on **c**). The arrow on b points some post-surgical blood products on the lateral wall of the right lateral ventricle. Note how the FLAIR hyperintensity extension increases on follow-up scans (arrowheads on **a,d,g**) after the onset of treatment with bevacizumab (BEV), while the enhancing area decreases dramatically (arrows on **f**), with reappearance despite antiangiogenic treatment (arrows on **i**).

As reviewed earlier, Macdonald criteria were initially criticized for considering the enhancing area of the tumor as the unique and absolute imaging biomarker of tumor response or progression. Then, with the advent of antiangiogenic therapy, the imperfections of this tool became more evident and rendered it invalid. The term *pseudoresponse* was designated to describe the decrease of the enhancement seen in the tumor (as much as to meet Macdonald's criteria for response) by the mere effect of the treatment with antiangiogenic drugs without a true antitumor effect (14, 26) (Figure 1). This phenomenon can be seen in up to 20–60% of patients receiving BEV and is attributed to its described stabilization effect on the BBB. Pseudoresponse was then considered as one of the most convincing explanations for the discrepancy between the astonishing response on MRI and limited overall survival rates.

Tumors usually build their vascular scaffold by using one of the following mechanisms: sprouting and branching from pre-existing vessels to form new capillaries (angiogenesis), *de novo* vasculogenesis from endothelial precursor cells, or utilization of mature vasculature after infiltrating normal host tissue (also called vessel co-option) (27). Further research demonstrated that when blocking angiogenesis with BEV, GBM's growth pattern changes and become more infiltrative, now privileging the vasculature co-option mechanism to meet its metabolic demands (28, 29). This growth pattern change is represented on the MRI as an increase of the extent of the non-enhancing part of the tumor, better appreciated as an expansion of the hyperintense areas on fluid-sensitive sequences (30). In 2009, Narayana et al. published the results of a descriptive study on sixty-one patients with recurrent high-grade gliomas that were treated with BEV (31). The main conclusion of this study was that BEV prolonged GBM patients' survival; however, the fascinating aspect of this study was that it served as one of the earliest reports on a possible increase of the aggressiveness of the tumor following antiangiogenic therapy, a topic that still is under active research (32, 33).

Regardless of the effectiveness of antiangiogenic molecules as antitumoral agents, it is noteworthy that their regulatory effect on the BBB entails a decrease of the vasogenic edema and mass effect exerted by the tumor, and it is translated into a slight improvement of the patients' symptoms and quality of life (21, 34). Conversely, the absence of pseudoresponse after the administration of BEV has been considered by some authors to be an ominous sign of worse prognosis (35).

From the clinical standpoint, patients with MRI results interpreted as pseudoresponse are often separated into two groups: symptomatic vs. asymptomatic. For the symptomatic group, immediate change of treatment regimen is usually the next step. For asymptomatic patients, continue the treatment received or continue observation with repeat MRI in 4–8 weeks are the frequent choice. Corticosteroid is usually offered to symptomatic patients while a new treatment regimen is implemented.

## Side Effects of Antiangiogenic Therapy on the Brain

BEV treatment has been reported to be safe and overall well-tolerated by patients with GBM in multiple trials (36); nonetheless, the most commonly mentioned side effects are fatigue, headache, hypertension, bowel perforation, and thromboembolism (37). Intracranial hemorrhage has been reported in <3% of patients on BEV (20, 38), and other reported CNS adverse effects include venous and arterial thromboembolic events and posterior reversible encephalopathy syndrome (PRES) (38, 39). Recent articles evaluating the clinical characteristics of BEV-associated ischemic and hemorrhagic strokes have pointed out that there is no significant difference when compared with control groups (40). The imaging manifestations of these entities do not differ from their appearance when caused by other etiologies.

## Current Status of Glioblastoma Response Assessment

### Response Assessment in Neuro-Oncology (RANO) Criteria

Taking into account the multifaceted nature of GBM treatment, OS may not accurately reflect the specific effect of new drugs or interventions in clinical trials, those being altered by SOC therapies used before or after the experimental one (1). Multiple clinical trials have used PFS and objective response rate (ORR) as indicators of response to the experimental therapeutic strategies (41). Consequently, it gives rise to the necessity of designing updated clinical and imaging criteria to quantify treatment response. To overcome these challenges, the Response Assessment in Neuro-Oncology (RANO) working group published the response assessment criteria for use in clinical trials of high-grade glioma (HGG) in 2010 (42) with an update on 2017 (43).

Currently, RANO workshop has published dedicated guidelines for high-grade gliomas (RANO-HGG), low-grade gliomas (RANO-LGG), brain metastasis (RANO-BM), leptomeningeal disease (RANO-LM), patients under immunotherapy (iRANO), spinal tumors (SPINO), meningioma (RANO-meningioma), and for pediatric tumors (RAPNO) (44). These guidelines standardize the MRI technique, scans schedule, measurement of lesions, clinical confounders for imaging findings, and response categories. Specifically, RANO-HGG considers CR when three criteria are met: (a) disappearance of all enhancing lesions sustained for at least 4 weeks, (b) the patient is off corticosteroids, and (c) the patient's clinical condition is stable or improving (43). With regards to the MRI scan, the first exam showing disappearance of all enhancing lesions will be considered as *preliminary CR*. If a second scan done 4 weeks apart still demonstrates disappearance of the enhancing lesions, then the patient will be further classified as *durable CR* and should continue the ongoing treatment. Conversely, if the follow-up exam re-demonstrates areas of enhancement, it will be considered pseudoresponse and the patient should be evaluated using the criteria of progressive disease. Notably, RANO-HGG incorporates the qualitative assessment of T2-FLAIR changes as an additional biomarker for tumor progression in the categories of PD, PR, and SD, making it stronger than the classic Macdonald criteria.

### The Role of Advanced Brain Tumor Imaging in Pseudoresponse

Current RANO-HGG criteria encompass both serial bi-dimensional/volumetric measurements of the enhancing portion of the tumor and the qualitative assessment of the infiltrative component detected by T2-FLAIR. Although it upgraded and superseded Macdonald criteria, it is still far from being a perfect tool to fully characterize the viability and spatial extension of GBM, specifically with regards to its invasive non-enhancing part. One of the most substantial frailties of RANO-HGG is its incapability of discriminating between non-enhancing infiltrative tumor and other causes of hyperintensity on T2-FLAIR such as vasogenic edema, microvascular ischemic changes, and other non-tumoral leukoencephalopathies. This is a limitation inherent to conventional MRI sequences which are pure anatomical imaging techniques; notwithstanding, researchers have developed some advanced MRI sequences that allow the functional assessment of tumors. Some of the advanced MRI sequences allow the evaluation of tumor perfusion, hemodynamics, chemistry and metabolism, and cellularity. Our institutional advanced brain tumor imaging (ABTI) protocol is run on 3T magnets and includes the aforementioned standard brain tumor protocol (conventional MRI sequences) and some advanced MRI sequences, whose applications on the evaluation of pseudoresponse will be briefly described in the following paragraphs.

**Figure 2:**
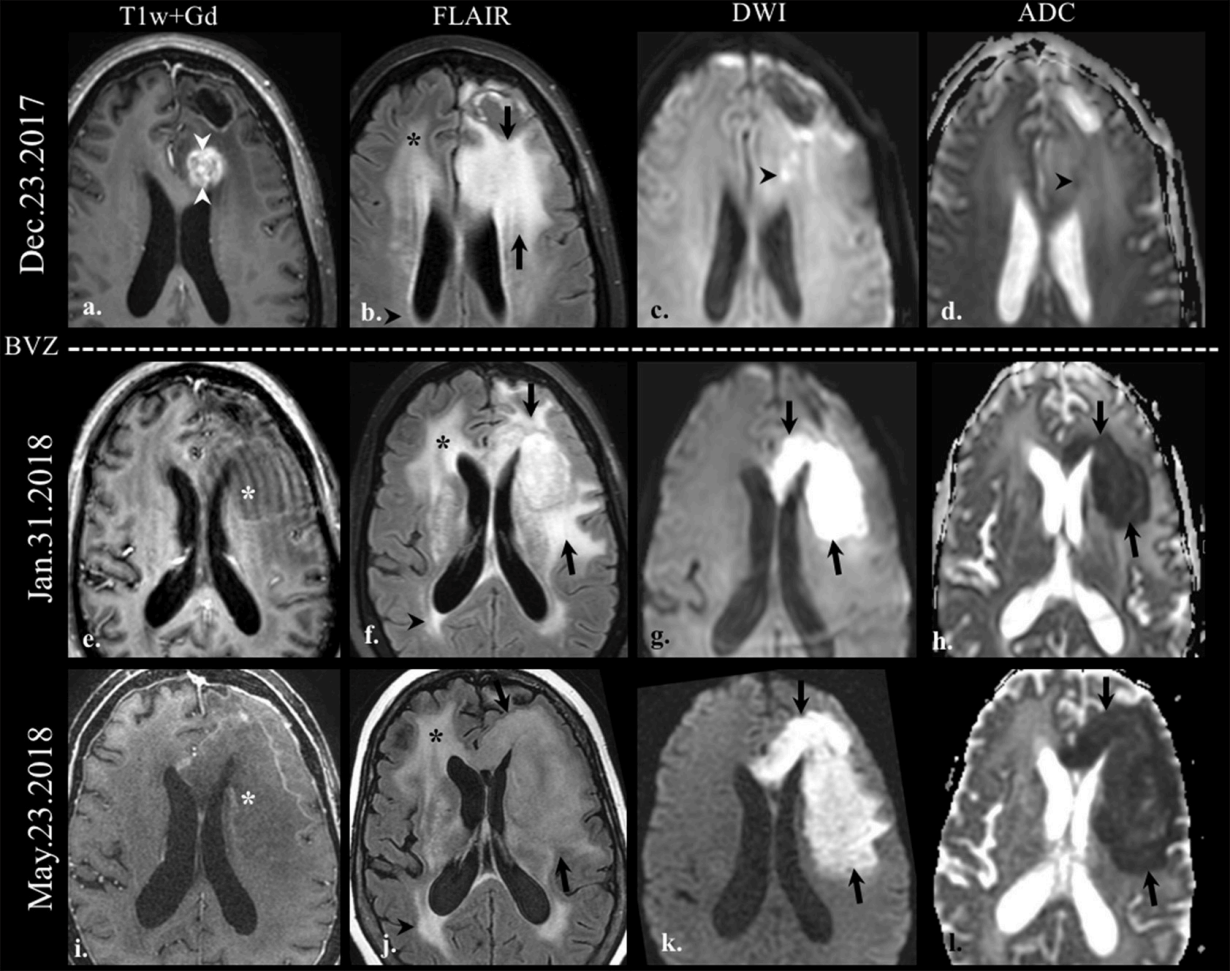
Diffusion-weighted imaging (DWI) in pseudoresponse. Brain MRI of a old patient with the history of GBM (IDH wild-type, MGMT status unknown) on the first recurrence. Axial gadolinium-enhanced T1w **(a,e,i)**, FLAIR **(b,f,j)**, DWI **(c,g,k)**, and apparent diffusion coefficient map **(d,h,l)** images are shown. The baseline scan (top row) shows FLAIR hyperintensity (arrows on **b**) involving the left frontal lobe adjacent to the posterior margin of the surgical cavity, along with an area of enhancement (arrowheads on **a**). There is a small focus of DWI hyperintensity (arrowhead on **c**) with its corresponding hypointensity on the ADC map (arrowhead on **d**) indicative of restricted diffusion. On follow-up scans after bevacizumab treatment (BEV) the enhancement disappeared (*on **e**,**i**); however, the FLAIR hyperintensity increased in size on the left cerebral hemisphere (arrows on **b,f,j**). Note the striking increase in the size of the area of restricted diffusion on follow-up scans (arrows on **g**,**k**, **h**,**l**) despite the absence of enhancement; this finding is indicative of the hypercellular nature of the tumor. There is non-specific progressive FLAIR hyperintensity on the right frontal lobe (*on **b,f,j**), and on the right periatrial region (arrowheads on **b,f,j**), without associated enhancement or restricted diffusion, likely treatment-related.

**Figure 3:**
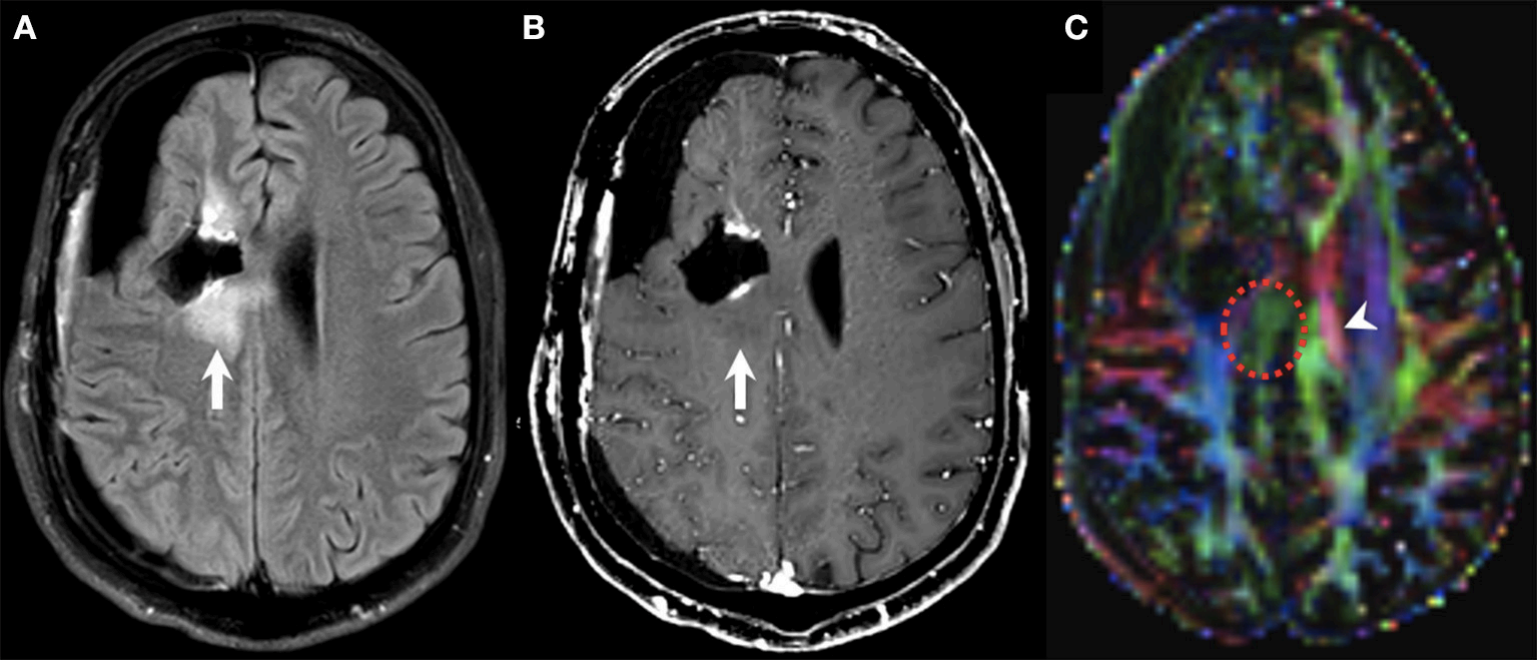
Diffusion tensor imaging (DTI) in pseudoresponse. Follow-up brain MRI of a 30-years-old male person with a diagnosis of GBM (NOS, MGMT status unknown) after standard-of-care treatment, currently on bevacizumab therapy. FLAIR **(A)**, contrast-enhanced T1w **(B)**, and DTI **(C)** axial images. A new area of non-enhancing FLAIR hyperintensity is noted along the posterior aspect of the right frontal surgical cavity (arrows on **A,B**) that was considered as infiltrative tumor based on other functional MRI sequences (not shown). DTI unveils the disruption of the corpus callosum fibers subjacent to the lesion (dotted circle on **C**) compared to the normal contralateral side (arrowhead on **C**) as a marker of tumor infiltration.

**Figure 4:**
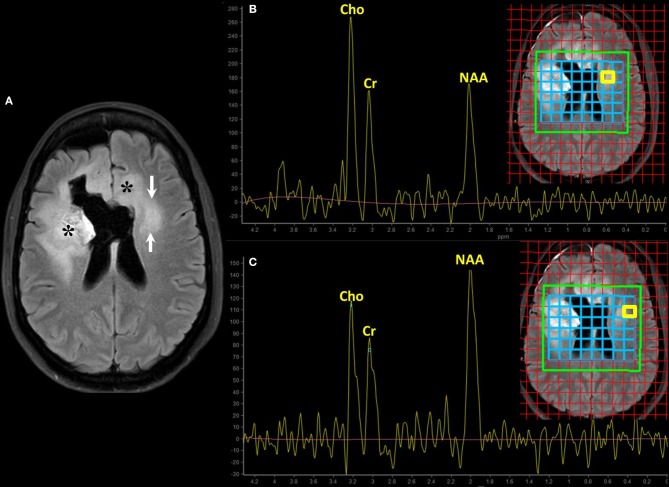
Magnetic resonance spectroscopy (MRS) for the assessment of cell proliferation. Brain MRI of a old patient with recurrent GBM (NOS, MGMT status unknown) on bevacizumab treatment (same patient of Figure 2, second row). Axial FLAIR **(A)** shows hyperintensity surrounding the surgical cavity on the right frontal lobe and in the contralateral periventricular white matter; asterisks (*) mark the enhancing areas seen on the previous scan. The clinical concern is to define whether the evolving area of FLAIR abnormality on the left side is vasogenic edema or tumor infiltration (arrows on **A**). Multivoxel MRS using intermediate echo time (144 ms) with interrogations on the region of interest **(B)** and the adjacent normal-appearing white matter **(C)** was performed. The spectrum corresponds to the area highlighted by the yellow box on the blue grid on the localizer images. The relative increase of choline (Cho) and the decrease in the N-Acetylaspartate (NAA) peak in the suspicious area compared to the normal-appearing one suggest high cell turnover rate and loss of neuronal integrity, respectively. These spectroscopic findings favor tumor infiltration over vasogenic edema.

**Figure 5:**
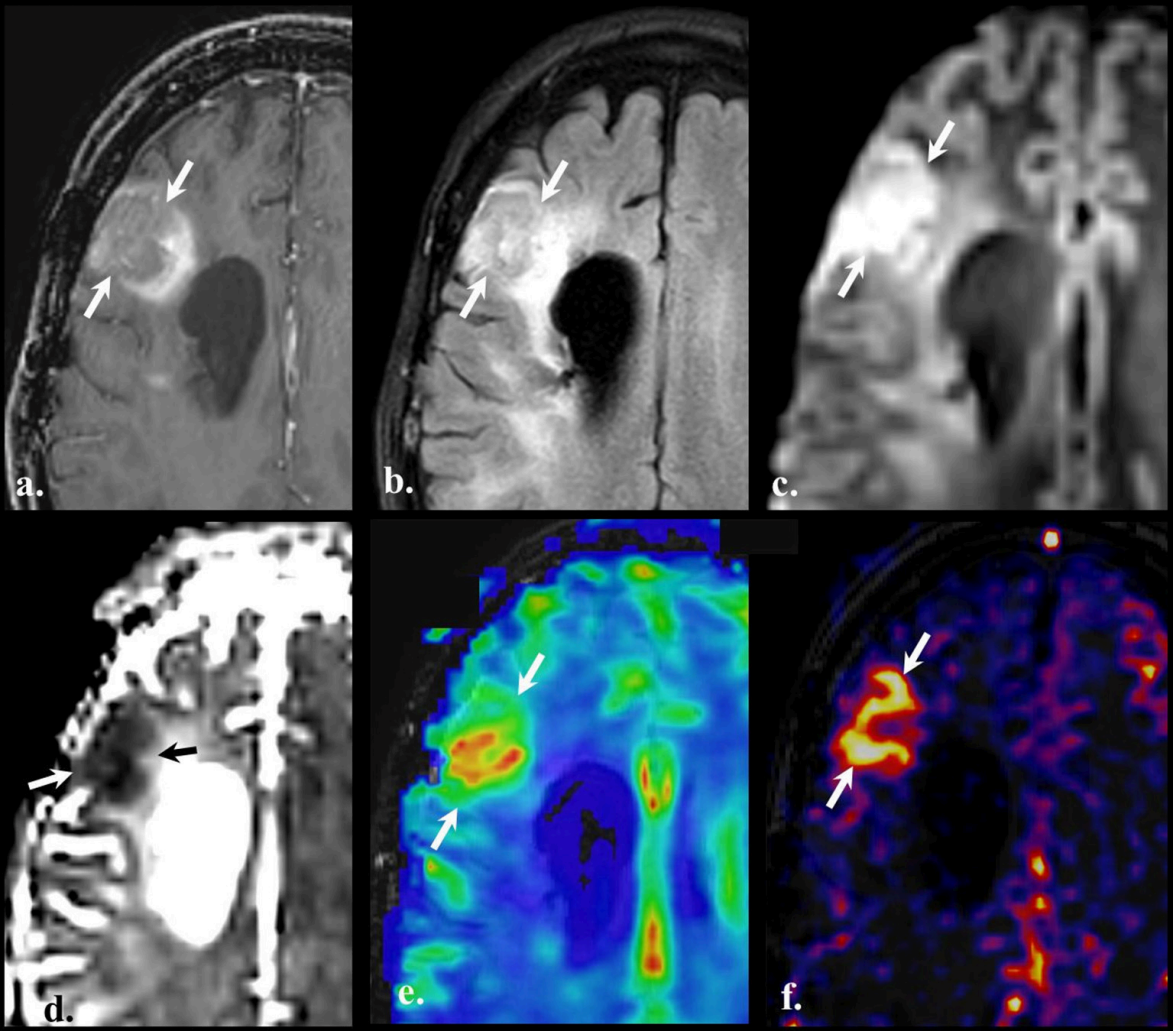
Perfusion-weighted imaging (PWI). Brain MRI of a old with a diagnosis of recurrent GBM (IDH wild-type, MGMT status unknown) on bevacizumab treatment. Axial contrast-enhanced T1w **(a)**, FLAIR **(b)**, DWI **(c)**, ADC **(d)**, Dynamic susceptibility contrast (DSC) **(e)**, and Pseudo-continuous arterial spin labeling **(f)**. Compared with the baseline scan, an evolving non-enhancing FLAIR hyperintense lesion (arrows on a and **b**) is noted on the right frontal lobe in the vicinity of the surgical cavity (not shown). The lesion shows high signal on DWI (arrows on **c**) with the reciprocal dark appearance on ADC map (arrows on **d**) due to hypercellularity. Dynamic susceptibility contrast depicts increased cerebral blood volume (CBV) (arrows on panel e), and pseudo-continuous arterial spin labeling (pCASL) reveals an increment in the cerebral blood flow in the same location. PWI findings are indicative of vascular proliferation.

- Diffusion weighted imaging
  There are two main types of water molecules in tissues, slow and fast diffusing. Slow-diffusing water molecules are attached to macromolecules or confined by cell membranes; conversely, fast-diffusing molecules are usually located in the extracellular space. Diffusion-weighted imaging (DWI) exploits this biochemical feature of water to characterize the composition of certain fluids (e.g., abscesses), evaluate the function of the Na+/K+-ATPase membrane pump in neurons (e.g., stroke), and in the neuro-oncology field, to estimate the cell density of tissues. The apparent diffusion coefficient (ADC) map is a representation of the magnitude of water diffusion restriction, with quantitative values expressed in units of mm^2^/s.
  In 1999, Sugahara et al. demonstrated that the cellular density and tumor grade of diffuse gliomas were directly proportional to the degree of water restriction on DWI (45). Additionally, in 2012 Yamasaki et al. published the results of a descriptive study on ten patients with recurrent high-grade glioma and stated that by using high magnetic field strengths, demonstration of water restriction on DWI is useful for the differentiation between pseudoresponse and true response to the antiangiogenic treatment (46). Furthermore, Kothari et al. demonstrated that DWI was superior in detecting tumor cell proliferation compared to FLAIR in patients with high-grade gliomas treated with BEV (47). Conversely, a recent retrospective study ran by Auer et al. reported a normalization of the mean ADC values in areas of pseudoresponse in patients with recurrent GBM under treatment with BEV (48). This result raises some concerns about the utility of this tool in the detection of the elusive infiltrative tumor. Regardless of the current uncertainty, this is a field of intense research, and for now, DWI can be considered as one of the ancillary tools for the detection of the invasive non-enhancing portion of the tumor in patients under antiangiogenic therapy (Figure 2).
  Diffusion tensor imaging (DTI) uses the anisotropy of the Brownian motion of water molecules caused by membranes to display the pathway of neural fibers *in vivo* (49). It is useful to evaluate the white matter tracts displacement, deformation, infiltration, disruption, or disorganization caused by tumors or other entities (50). The fraction of anisotropy (FA), axial diffusivity (AD), and radial diffusivity (RD) are some of the quantitative metrics derived from diffusion tensor imaging, and have been used to characterize and classify CNS tumors (51–53); however, its role on the assessment of pseudoresponse is not well-established to date (Figure 3).
- MR spectroscopy
  The MR spectroscopy (MRS) is a technique that uses the carbon-bound hydrogens in the −1 to −5 ppm range of the chemical shift scale to determine the presence and concentration of certain molecules in a given sample of brain tissue. The most commonly measured metabolites include N-acetylaspartate (NAA), choline (Cho), creatine (Cr), myo-inositol (mIns), lactate, lipids, and certain amino acids (54). In glial neoplasms, the higher the Cho, the higher the tumor grade is; and the NAA and creatine levels are inversely proportional to the aggressiveness of the tumor. Some studies have shown the utility of MRS in the non-invasive assessment of glial tumors grading (55, 56), assessment of tumor extension (57), and in the differentiation of pseudoprogression vs. recurrent/residual tumor (58, 59).
  As mentioned previously, the rise of Cho is a strong predictor of rapid cell turnover and tumor cell replication. Ratai et al. evaluated 13 patients with recurrent GBM on BEV in combination with either temozolomide or irinotecan and demonstrated that variations in NAA and Cho peaks on MRS could be used as a reliable imaging biomarker for discriminating between response and pseudoresponse in patients under antiangiogenic therapy (60). Hattingen et al. described the utility of non-conventional metabolites on *in-vivo* MRS for the assessment of the effectiveness of BEV in patients with recurrent GBM. Although the trial showed promising results, these MRS techniques are not available in most of the non-academic healthcare facilities (61). Possibly, the most practical utility of MRS in the daily radiological practice is for the differentiation between vasogenic edema vs. non-enhancing tumor infiltration using an array of multivoxel spectroscopy either in a 2D or 3D acquisition (62) (Figure 4).
- Perfusion-weighted imaging
  Perfusion-weighted imaging (PWI) is the name given to multiple MR sequences designed to evaluate brain hemodynamics at the capillary level. Dynamic susceptibility contrast (DSC) is one of the MR techniques used for the evaluation of brain blood perfusion. The physical foundation of this MR sequence relies on the amount of signal loss on T2^*^ weighted sequences elicited by the pass of a bolus of a GBCA through a capillary bed. The most commonly calculated measurements are mean transit time (MTT), cerebral blood flow (CBF), and cerebral blood volume (CBV). Those measures are representatives of the time that red blood cells spend, the volume of blood passing per unit of time, and the volume of blood in a given amount of brain tissue, respectively (63). As previously stated, one of the histologic landmarks of some of the most common malignant CNS tumors is the elevated rate of angiogenesis. The abnormal vascular proliferation increases the amount of blood per brain tissue volume unit and, consequently, it increases the relative CBV values in PWI (64, 65). Research showed that the difference of the relative CBV (rCVB) values before and after treatment of recurrent GBM with BEV serves as a biomarker of treatment response, and patient prognosis (66, 67).
  On the other hand, the pseudo-continuous arterial spin labeling (pCASL) is a perfusion-weighted imaging (PWI) technique that does not require intravenous administration of GBCA. pCASL exploits the ability of MRI scans of magnetically labeling arterial blood protons below the level of the imaging plane (68). In clinical practice, pCASL provide some quantitative and qualitative measurements of blood hemodynamics in brain tissue such as blood volume, blood velocities, and blood transit times (69). There is substantial evidence supporting that measurements obtained from DCS and pCASL techniques are comparable and their diagnostic performance is similar (70, 71). The most notable metric in pCASL is CBF, being the tumor blood volume directly proportional with the tumor grade (72, 73). It is noteworthy that even when PWI relies on the degree of vascularity of the tissue, it is not strictly coupled with the area of visible enhancement. Price et al. demonstrated that perfusion altered metrics extends beyond the limits of the area of enhancement in high-grade gliomas (74). Experimental studies have shown that normalized CBV and normalized CBF obtained from DSC are comparable with normalized CBF on arterial spin-labeling and are useful for the assessment of the treatment effect in patients with recurrent GBM under antiangiogenic therapy (75, 76) (Figure 5).
- Susceptibility weighted imaging
  Susceptibility weighted imaging (SWI) is an MRI sequence that utilizes the difference in the intrinsic magnetic susceptibility of tissues as an endogenous source of image contrast (77). One of the most clinically relevant utilities of SWI is its ability to identify small amounts of iron-containing molecules or calcium, knowing that both of them may be non-visible on conventional MR sequences. The SWI has been used in the clinical setting in many ways, including the study of vascular lesions, due to its exceptional resolution in depicting venous anatomy (78), and the evaluation of multiple sclerosis (79, 80). Additionally, some authors have described the utility of SWI for discriminating between low and high-grade gliomas by characterizing the intratumoral susceptibility signal (81, 82). Lupo et al. ran a descriptive study on twenty-five patients with GBM before and after treatment with chemoradiotherapy plus BEV (83). The researchers performed a baseline MRI scan before, and serial examinations after treatment until the date of the first recurrence, and found that the more crowded and widespread the foci of susceptibility artifact inside the lesion, the less likely to find viable tumor underneath. Experimental studies have shown that with ultra-high-field MRI, SWI can demonstrate even the growing vasculature in the tumor and its change upon treatment with BEV (84); however, it remains in the research arena.

## Challenges for the Oncologic Neuroradiology Team

Currently, BEV is one of the most commonly used treatment options for recurrent GBM, either alone or with other drugs. However, there are conflicting results in the literature about its antitumor efficacy vs. “super steroid” effect, commonly referred to as pseudoresponse since there is a reduction of enhancement on CT or MRI scans post BEV therapy. We cannot resolve this issue in this review, but we feel that a combination of chemotherapeutic drugs including BEV may provide true anti-tumor effect (85). From an imaging perspective, it is challenging to report such phenomenon during daily clinical practice and for assessment of effects of any clinical trial on recurrent GBM involving BEV therapy. Therefore, clinical information, such as the history of BEV based therapy is vital for more accurate reporting for neuro-radiologist. Advanced imaging tools mentioned above are hopeful to provide a more accurate assessment of treatment effect. Also, new genetic-based classification of tumors creates subsets of patients that may have different response patterns to immune therapy drugs, such as humanized monoclonal antibody against the cytotoxic T-lymphocyte antigen-4 (CTLA-4) immune checkpoint, and against the programmed death 1 (PD-1) receptor and its ligand PD-L1 (84, 86). This scenario gives rise to the need to design imaging protocols that merge the anatomical and functional imaging biomarkers on light of the new therapies. Possibly, the most significant challenges are to find imaging biomarkers to typify the antitumor effect of BEV (cellularity and cell replication); to identify and map the shift toward an infiltrative growth pattern; to establish key imaging findings that define prognosis; and to detect early BEV complications.

## Conclusion

GBM remains as one of the deadliest malignancies affecting the CNS despite long-standing research efforts to improve the treatment options for these patients. Developing better imaging tools to evaluate the therapeutic effect of different interventions in real time will have a direct impact on the process of developing new and more effective treatments. Many revised standardization of brain tumor assessment have been published during the past decades; however, the relentless expansion of novel treatments and neuroimaging techniques render them outdated very quickly. Currently, RANO-HGG criteria are the standard for assessment of treatment response; nonetheless, it is still considered a work in progress while some advanced MRI techniques become more widely available, standardized, and reproducible. Advanced MRI sequences provide valuable information about functional tumor variables, such as cellularity, biochemistry, metabolism, cell turnover estimation, vascularization, and microvascular hemodynamics that can help the examiner to track the presence of non-enhancing tumor in patients with recurrent GBM under antiangiogenic treatment.

## Author Contributions

All authors contributed to the literature research, manuscript design and writing, image edition and submission equally.

### Conflict of Interest Statement

The authors declare that the research was conducted in the absence of any commercial or financial relationships that could be construed as a potential conflict of interest.

## Abbreviations

3D-T1w: Three-dimensional T1-weighted images
ABTI: Advanced brain tumor imaging
ADC: Apparent diffusion coefficient
BBB: blood-brain barrier
Cho: Choline
BEV: bevacizumab
CBF: Cerebral blood flow
CNS: Central nervous system
CR: Complete response
Cr: Creatine
CT: Computerized tomography
DSC: Dynamic susceptibility contrast
DWI: Diffusion-weighted imaging
FLAIR: T2-Fluid-attenuated inversion recovery
GBCA: Gadolinium-based contrast agent
GBM: Glioblastoma
HGG: High-grade glioma
IDH: Isocitrate dehydrogenase
MGMT: O^6^-methylguanine-DNA-methyltransferase
mIns: Myo-inositol
MRI: Magnetic resonance imaging
MRS: Magnetic resonance spectroscopy
MTT: Mean transit time
NAA: N-acetylaspartate
ORR: Objective response rate
OS: Overall survival
pCASL: Pseudo-continuous arterial spin labeling
PD: Progressive disease
PFS: Progression-free survival
PR: Partial response
PWI: Perfusion-weighted imaging
RANO: Response Assessment in Neuro-Oncology
rCBV: Relative cerebral blood volume
SD: Stable disease
SOC: Standard-of-care
SWI: Susceptibility weighted imaging
T2w: T2-weighted
TTF: Tumor treating fields therapy
VEGF: Vascular endothelial growth factor
WHO: World Health Organization.
